# A Quantum-Enhanced Precision Medicine Application to Support Data-Driven Clinical Decisions for the Personalized Treatment of Advanced Knee Osteoarthritis: The Development and Preliminary Validation of precisionKNEE_QNN

**DOI:** 10.7759/cureus.52093

**Published:** 2024-01-11

**Authors:** Nima Heidari, Stefano Olgiati, Davide Meloni, Mark Slevin, Ali Noorani, Federico Pirovano, Leonard Azamfirei

**Affiliations:** 1 Medical Supercomputation and Machine Learning, European Quantum Medical, London, GBR; 2 Foot, Ankle and Limb Reconstruction, Orthopaedic Specialists, London, GBR; 3 Medicine, Pharmacy, Science and Technology, George Emil Palade University, Targu Mures, ROU; 4 Medical Supercomputation and Biostatistics, European Quantum Medical, Milan, ITA; 5 Department of Biomedical Technologies and Translational Medicine, University of Ferrara, Ferrara, ITA; 6 Supercomputation and Artificial Intelligence, European Quantum Medical, Turin, ITA; 7 Upper Limb, Orthopaedic Specialists, London, GBR; 8 Quantum Machine Learning, European Quantum Medical, Milan, ITA

**Keywords:** quantum machine learning, biologic therapies, personalised precision medicine, joint preservation, osteoarthritis knee

## Abstract

Background

Quantum computing and quantum machine learning (QML) are promising experimental technologies that can improve precision medicine applications by reducing the computational complexity of algorithms driven by big, unstructured, real-world data. The clinical problem of knee osteoarthritis is that, although some novel therapies are safe and effective, the response is variable, and defining the characteristics of an individual who will respond remains a challenge. In this study, we tested a quantum neural network (QNN) application to support precision data-driven clinical decisions to select personalized treatments for advanced knee osteoarthritis.

Methodology

After obtaining patients’ consent and Research Ethics Committee approval, we collected the clinicodemographic data before and after the treatment from 170 patients eligible for knee arthroplasty (Kellgren-Lawrence grade ≥3, Oxford Knee Score (OKS) ≤27, age ≥64 years, and idiopathic aetiology of arthritis) treated over a two-year period with a single injection of microfragmented fat. Gender classes were balanced (76 males and 94 females) to mitigate gender bias. A patient with an improvement ≥7 OKS was considered a responder. We trained our QNN classifier on a randomly selected training subset of 113 patients to classify responders from non-responders (73 responders and 40 non-responders) in pain and function at one year. Outliers were hidden from the training dataset but not from the validation set.

Results

We tested our QNN classifier on a randomly selected test subset of 57 patients (34 responders, 23 non-responders) including outliers. The no information rate was 0.59. Our application correctly classified 28 responders out of 34 and 6 non-responders out of 23 (sensitivity = 0.82, specificity = 0.26, F1 Statistic = 0.71). The positive and negative likelihood ratios were 1.11 and 0.68, respectively. The diagnostic odds ratio was 2.

Conclusions

Preliminary results on a small validation dataset showed that QML applied to data-driven clinical decisions for the personalized treatment of advanced knee osteoarthritis is a promising technology to reduce computational complexity and improve prognostic performance. Our results need further research validation with larger, real-world unstructured datasets, as well as clinical validation with an artificial intelligence clinical trial to test model efficacy, safety, clinical significance, and relevance at a public health level.

## Introduction

Applied research has shown that quantum computing applied to machine learning is a novel experimental digital medicine technology [1] which can reduce computational complexity [2,3] and improve data-driven clinical decisions [4].

Several biologic therapies, including the injection of microfragmented adipose tissue, have been shown to be safe and effective [5-7]. The outcomes of these treatments can be variable [8], with some patients showing dramatic improvements in their symptoms while others failing to respond [9].

Defining the characteristics of individuals who will respond remains a challenge. Applications and tools to support clinical decisions with predictive, data-driven, precision medicine can identify patients who will respond with reduction in pain and improvement in function to novel as well as established treatments based on pre-treatment clinicodemographic data [9]. This approach is key in developing personalized, evidence-based clinical pathways.

An earlier version of this article was previously posted to the medRxiv preprint server in 2021 [10].

## Materials and methods

Ethics statement

This study was conducted in compliance with the rules of the Helsinki Declaration and International Ethical Regulations [11], including all subsequent amendments, under the approval of the Research Ethics Committee of the George Emil Palade University of Medicine, Pharmacy, Science and Technology of Targu Mures, Romania (approval number: 1464/2021).

Dataset

After obtaining patients’ consent and Research Ethics Committee approval, we collected clinicodemographic data before and after the treatment from 170 patients eligible for knee arthroplasty (Kellgren-Lawrence grade ≥3, Oxford Knee Score (OKS) ≤27, age ≥64 years, and idiopathic aetiology of arthritis) treated over a two-year period with a single injection of microfragmented fat (Table 1). Gender classes were balanced (76 males and 94 females) to mitigate gender bias. A patient with an improvement ≥7 OKS was considered a responder.

**Table 1:** Baseline data. Age on procedure, preoperative Oxford Knee Score (OKS), and Kellgren-Lawrence (KL) grade of knee osteoarthritis. Source dataset 1464/2021.

	Mean ± SD	Range (minimum–maximum)
Age	74 ± 7	65–92
Preoperative OKS	21 ± 6	18–27
KL grade	4 ± 0.4	3–4

Oxford Knee Score

OKS comprises 12 questions that are scored 0-4, with 0 being severe compromise and 4 no compromise, covering pain and function of the knee. The best outcome is a score of 48 and the worst score possible is 0. This is a validated score or a measure of functional outcomes in patients undergoing knee arthroplasty. All 170 patients completed questionnaires before and at three months, six months, and one year following treatment [12].

Kellgren-Lawrence classification of the severity of osteoarthritis

The Kellgren-Lawrence system is a common method of classifying the severity of osteoarthritis using the following five grades [13]: (1) grade 0 (none): definite absence of X-ray changes of osteoarthritis; (2) grade 1 (doubtful): doubtful joint space narrowing and possible osteophytic lipping; (3) grade 2 (minimal): definite osteophytes and possible joint space narrowing; (4) grade 3 (moderate): moderate multiple osteophytes, definite narrowing of the joint space, and some sclerosis and possible deformity of bone ends; and (5) grade 4 (severe): large osteophytes, marked narrowing of the joint space, severe sclerosis, and definite deformity of bone ends. Osteoarthritis is deemed present at grade 2 although of minimal severity 1.

Model and circuit description

**Figure 1:**
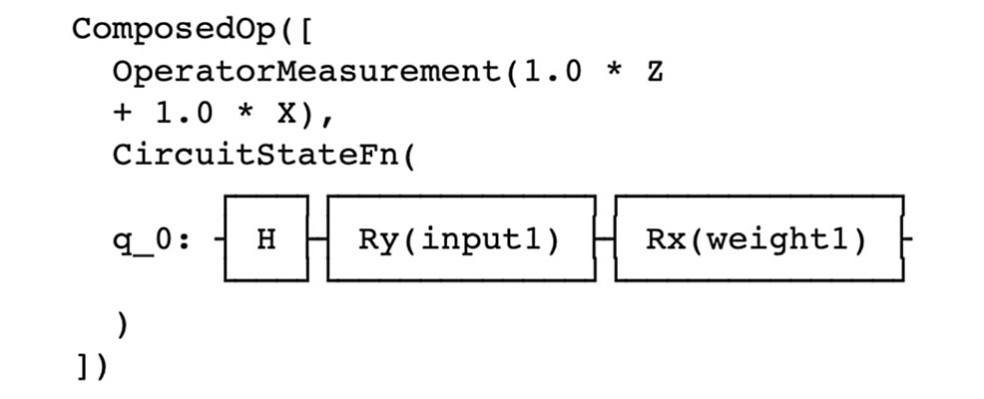
Circuit description. Source: IBM, Qiskit, and OpflowQNN. Copyright notice: our code is an alteration of Qiskit Machine Learning which is a Copyright of IBM 2017, 2021. Qiskit is licensed under the Apache License, Version 2.0. All modifications of the code and derivative works are the responsibility of the authors. q 0 = qubit initialization; Z, X = Pauli Z and X gates; H = Hadamard gate; Ry = single-qubit rotation gate through angle θ (radians) around the y-axis; Rx = single-qubit rotation gate through angle θ (radians) around the x-axis

We applied the approach to quantum problem-solving developed by Zickert [14]. We utilized a Parameterized Quantum Circuit (PQC) based on Quantum Machine Learning Neural Networks Classifier (QNN) technology developed by IBM and Qiskit Machine Learning. More specifically, we utilized OpflowQNN which is a neural network-based application for the evaluation of quantum mechanical observables. The OpflowQNN circuit is described in Figure 1.

Model training

**Figure 2:**
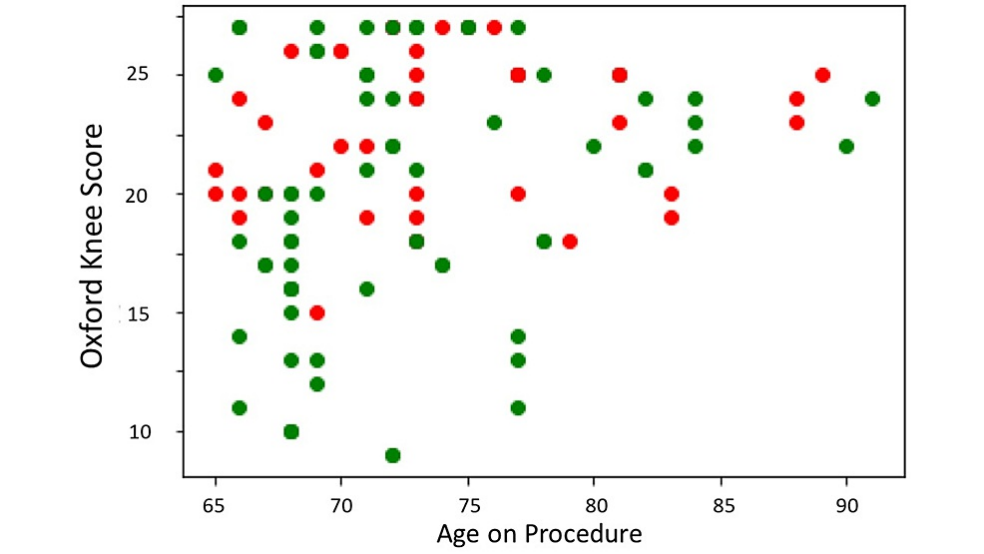
Training dataset. X-axis = age on procedure; Y-axis = preoperative Oxford Knee Score; Green dots = responders with an improvement of ≥7 points in the Oxford Knee Score at year one; Red dots = non-responders with an improvement of <7 points in the Oxford Knee Score at year one. Source Dataset 1464/2021.

We trained our QNN classifier on a randomly selected training subset of 113 patients treated over a two-year period with a single injection of microfragmented fat classified as responders (R; OKS improvement ≥7) and non-responders (NR; OKS improvement <7) in pain and function at one year (73 R, 40 NR). Outliers were hidden from the training dataset but not from the validation set (Figure 2).

**Figure 3:**
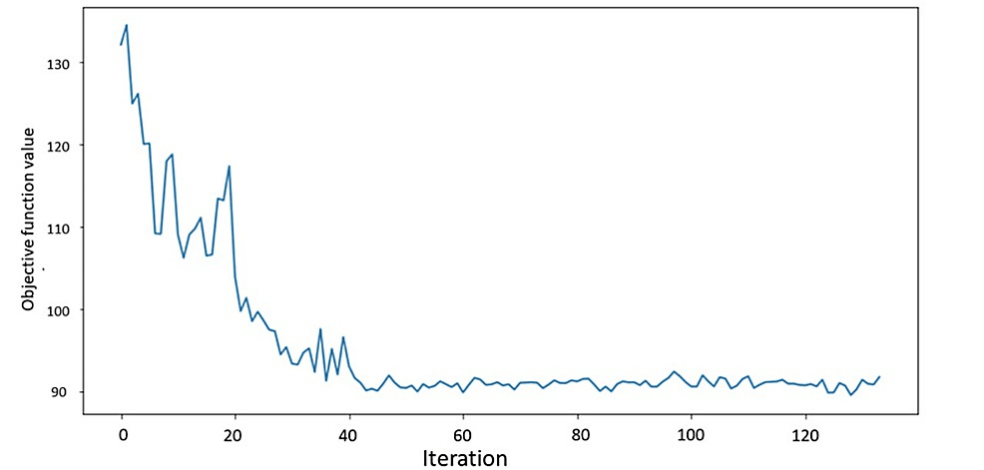
Objective function value against Iteration. Constrained Optimization BY Linear Approximation11 (COBYLA) optimizer to minimize the objective function value. Source: IBM, Qiskit, and OpflowQNN.

We initialized a 2-qubit Aer Simulator Backend with 1,024 shots. We utilized a Constrained Optimization BY Linear Approximation (COBYLA) optimizer to minimize the objective function value (Figure 3).

Model validation

**Figure 4:**
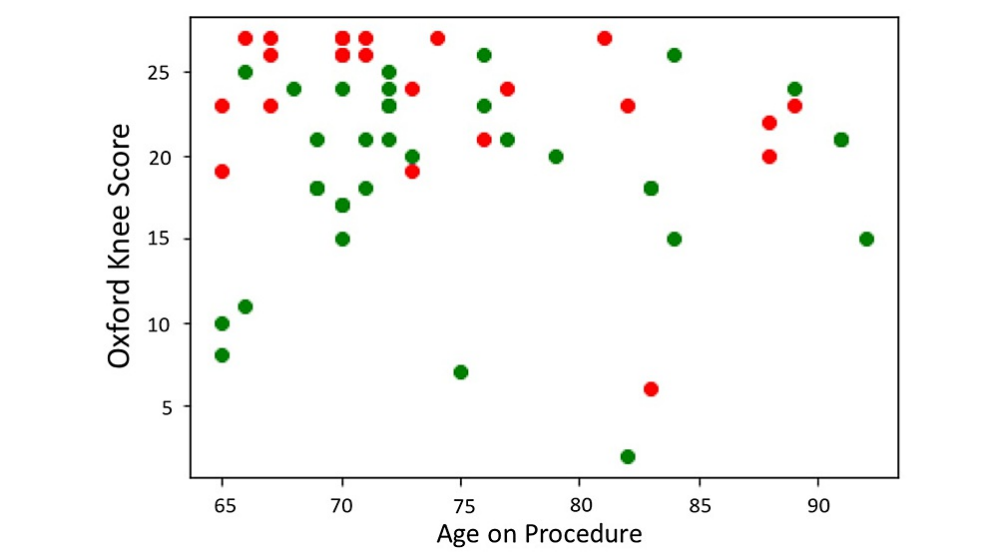
Test dataset. X-axis = age on procedure; Y-axis = preoperative Oxford Knee Score; Green dots = responders with an improvement of ≥7 points in the Oxford Knee Score at year one; Red dots = non-responders with an improvement of <7 points in the Oxford Knee Score at year one. Source Dataset 1464/2021.

The application was tested on a balanced raw dataset including outliers of 57 patients (34 R, 23 NR) with Kellgren- Lawrence grades of 3 and 4, OKS ≤s27, age ≥64 years, and idiopathic aetiology of arthritis (Figure 4, Table 2).

**Table 2:** Test performance metrics. Source precisionKNEE QNN applied to the test set from dataset 1464/2021. LR+ = positive likelihood ratio; LR- = negative likelihood ratio; DOR = diagnostic odds ratio

Metric	Value
Sensitivity	0.8235
Specificity	0.2609
LR+	1.1142
LR-	0.6765
DOR	2.0000
F1	0.7088

## Results

Model results

**Figure 5:**
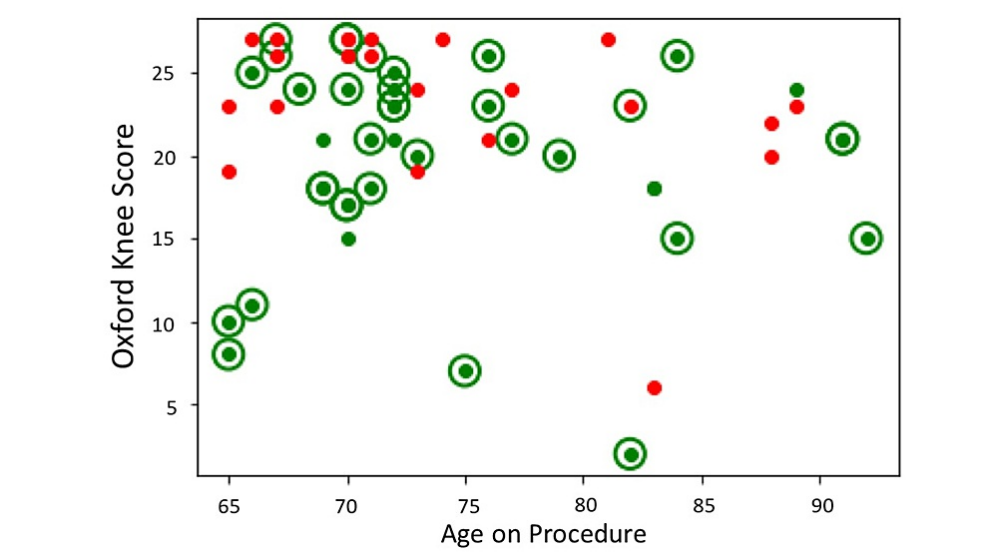
Classification with precisionKNEE_QNN. X-axis = age on procedure; Y-axis = preoperative Oxford Knee Score; Green dots = responders with an improvement of ≥7 points in the Oxford Knee Score at year one; Red dots = non-responders with an improvement of <7 points in the Oxford Knee Score at year one; Green circles = correct classification of either responders or non- responders; No circles = incorrect classification of either responders or non-responders. Source dataset 1464/2021.

Our application correctly classified 28 responders out of 34 and 6 non-responders out of 23 (sensitivity = 0.82, specificity = 0.26, F1 statistic = 0.71). The positive (LR+) and negative (LR-) likelihood ratios were 1.11 and 0.68, respectively. The diagnostic odds ratio was 2 (Table 3, Figure 5).

**Table 3:** Confusion matrix. Source precisionKNEE QNN applied to the test set from dataset 1464/2021. R = responders; NR: non-responders

		Predicted		
		R	NR	Observed
Observed	R	28	6	34
	NR	17	6	23
	Predicted	45	12	57

Model interpretation

The use of the precisionKNEE QNN clinical decision-making tool requires external validation. This is a new technology best suited to large unstructured datasets. Some of the traditional methods of validation may not be appropriate for quantum machine learning tools such as ours [15].

The SPIRIT-AI [16], and CONSORT-AI [17] initiatives provide guidance to improve the transparency and completeness of reporting clinical trials evaluating interventions involving artificial intelligence. The utility of these initiatives is not clear for use in quantum machine learning.

## Discussion

Since its original proposal by Richard Feinman [18], quantum computing has evolved from a concept to a practical technology [19] which is now being actively utilised with the potential to solve some of the more complex problems. These probably fall into the following three categories: (1) simulating nature - including chemistry, materials science, and physics; (2) processing data with a complex structure - including artificial intelligence/machine learning (AI/ML), factoring, and ranking; and (3) search and optimization - including pricing, risk analysis, and sampling [20].

A recent detailed review of quantum computer literature associated with the medical sciences [20] detailed three broad areas that most studies fit into, which include genomics and clinical research, diagnostics, and treatments and interventions.

Our study falls into the treatments and interventions category where we tested a QNN application to support precision data-driven clinical decisions to select personalized treatments for advanced knee osteoarthritis. QNNs were applied to clinicodemographic data from 170 individuals who were treated with microfragmented fat and followed up for over two years. In the assessment of the test’s diagnostic accuracy (Table 2), it was found to have a sensitivity of 82.35%, indicating a reasonably high ability to detect true positives. However, its specificity was notably low at 26.09%, suggesting a substantial rate of false positives. The LR+ of 1.1142 and LR- of 0.6765 further indicated that the test was only marginally more effective than chance in distinguishing between the presence and absence of the condition. The DOR was 2.0, reinforcing the test’s limited discriminative power. Despite these limitations, the F1 score of 0.7088 showed a moderate balance between precision and recall, reflecting an average overall accuracy in the test’s performance.

Healthcare practices today often rely on inferring insights from extensive datasets and applying these to individual cases. This approach is more refined than historical methods but still falls short of providing personalized treatment for each unique patient. The intricate nature of human biology means that medical interventions typically contribute just 10% to 20% to health outcomes. The remaining 80% to 90% is influenced by factors such as lifestyle choices, socioeconomic status, and environmental conditions [21].

There are many intersectional challenges at the nexus of quantum computing and healthcare, which will necessitate the refinement of quantum algorithms to adeptly manage complex datasets characterized by high dimensionality and variability [22], while simultaneously striving to minimize computational errors through enhanced physical devices and sophisticated software techniques. A pivotal goal is to expand quantum computational resources by increasing the available qubits, thereby enriching the capacity to encode complex features.

In tandem with these quantum-specific challenges, digital health strategies must prioritize transparent model explainability to demystify AI decision-making processes, ensuring that patient sovereignty over personal health data is uncompromised. There are indeed a multiplicity of technical and ethical challenges to the full deployment of quantum-enhanced health and medicine. This challenge has been taken out by the Cleveland Clinic and their collaboration with IBM to embed quantum computation into the workflow of providing personalised precision medicine [23].

Alongside modern technologies, it is important to recognise that there are broad and well-established principles that must be borne in mind when aiming to improve healthcare with the simultaneous pursuit of three aims, namely, improving the experience of care, improving the health of populations, and reducing per-capita costs of healthcare [24]. The more recent addition of the health and well-being of healthcare professionals [25] is a testament to how integral these individuals are in the delivery of healthcare as well as the innovation required to deliver the highly technological aspects of personalised precision medicine.

Limitations

Despite being one of the largest datasets of its kind, upon stratification we observed groups of patients with minimal observations and thus we cannot conclude external validity in the findings pertaining to these groups. Kellgren-Lawrence classification of arthritis was found to be an important feature during modelling. This measure is crude and correlates poorly with patient symptoms.

Microfragmented fat was the only treatment used in our model. With the inclusion of other treatment modalities, a true clinical decision-making support tool can be developed. Patients find these very useful and by providing a personalised prediction of the possible outcome, they will be able to play a more meaningful role in the decision-making process.

## Conclusions

Preliminary results on a small validation dataset show that quantum machine learning applied to data-driven clinical decisions for the personalized treatment of advanced knee osteoarthritis is a promising technology to reduce computational complexity and improve prognostic performance.

Our results need further research validation with larger, real-world unstructured datasets, and clinical validation with an AI clinical trial to test model efficacy, safety, clinical significance, and relevance at a public health level.
